# Practice of Routine Monitoring of Gastric Residual in Preterm Infants: A Meta-Analysis Article

**DOI:** 10.3390/children12040526

**Published:** 2025-04-20

**Authors:** Hassan Al-shehri

**Affiliations:** Department of Pediatrics, College of Medicine, Imam Mohammad Ibn Saud Islamic University (IMSIU), Riyadh 13317, Saudi Arabia; haalshehri@imamu.edu.sa; Tel.: +966-112-037100 (ext. 502)

**Keywords:** feed intolerance, gastric aspirates, gastric residual, low birth weight, necrotizing enterocolitis, preterm

## Abstract

Background: Controversy exists about the usefulness of gastric residual (GR) evaluation in preterm infants, and different results have been obtained in studies addressing this practice. Therefore, this meta-analysis aimed to evaluate the efficacy and safety of the practice of routine monitoring of GR compared to avoiding routine aspiration or alternative interventions. Methods: An online database search was conducted for relevant randomized trials from 2017 to 2023. The efficacy of the intervention was assessed from the incidence of necrotizing enterocolitis (NEC) and the time taken for full enteral feeds. The safety was assessed from the duration of hospitalization, incidence of late-onset sepsis, and days of total parenteral nutrition. Results: Only six studies were deemed eligible, fit the inclusion criteria, and were included in the quantitative synthesis. There was no significant difference between the groups in the incidence of NEC, with a mean difference of 0.95 (95% CI: 0.52, 1.75), while the intervention practice showed the early achievement of full enteral feeds (−2.21; 95% CI: −2.58, −1.84), a shorter duration of hospitalization (−0.65; 95% CI: −1.33, 0.02), a lower incidence of late-onset sepsis (0.70; 95% CI: 0.45, 1.09), and less days of total parenteral nutrition −1.65 (95% CI: −1.90, −1.40). Conclusions: For preterm infants with no signs of feeling intolerance, the results from this study stress the omission of the practice of routine gastric residual aspiration.

## 1. Introduction

Gastric residual (GR) evaluation, the traditional and primary tool utilized by neonatologists to evaluate feeding intolerance in preterm neonates, is involved in predicting late short-term nutritional outcomes and clinical situations such as feeding intolerance, necrotizing enterocolitis, and severe infections, allowing clinicians to preempt the risk of significant adverse outcomes in these unstable neonates [1,2]. The recommendation for the evaluation of GR in clinical practice is found in a series of reviews and clinical guidelines, but in general, the use of this method is limited due to compliance and the volume of the aspirated content [3,4,5].

Premature infants frequently exhibit delayed oral feeding abilities due to challenges in coordinating a suitable suck–swallow–breathe pattern, which is essential for achieving full oral feedings. Inability to attain complete oral feedings promptly results in numerous short- and long-term complications for the newborn, caregivers, and the healthcare system. Early nutrition is one of the mainstays of routine care for very preterm infants. Adequate nutrition improves long-term development and increases muscle and fat mass. However, preterm infant feeding is complex, and there are still many challenges, including difficulties in achieving adequate enteral nutrition. Necrotizing enterocolitis, alterations in the gastroduodenal function, and increased length of stay in the neonatal intensive care unit are some of the extra-uterine growth failure risk factors [6,7,8]. Accordingly, the worldwide initiative of the neonatal feeding operation is included in clinical practice guidelines with the goal of preventing complications related to feeding intolerance, and which demands close attention to the capability of preterm feeding rather than only providing sufficient nutrients [9].

Based on this practice of gastric residual evaluation, healthcare practitioners may decide on feeding management in different ways and may lack uniform criteria, which might result in inconsistent feeding management judgments. This discrepancy could cause unwarranted delays in the initiation or progression of feeds, which could lead to an extended utilization of total parenteral nutrition (TPN) [10] and an increased risk of liver illness associated with parenteral nutrition [11]. Due to delayed enteral feeding, the prolonged use of TPN may require prolonged use of a central venous line (CVL), which entails additional hazards, such as a higher chance of late-onset sepsis [12]. It is of utmost importance for all neonatal critical care units to prevent central line-associated bloodstream infections (CLABSIs) [13].

To guarantee that assessing GR in preterm newborns is a widely accepted routine clinical practice, its utility and efficacy as a prophylactic tool against necrotizing enterocolitis (NEC) or aspiration pneumonia must be established and validated from time to time. Thus, the objective of this study was set to evaluating the efficacy and safety of the practice of GR evaluation for preterm babies compared to avoiding the practice or using alternative procedures. The purpose of this meta-analysis was to answer the following research question bounded by the PICO format: “Does the use of GR volume evaluations compared to placebo influence the clinician’s decisions on preterm infant feeds?”.

## 2. Materials and Methods

### 2.1. Protocol

This review study was pre-registered with the International prospective register of systematic reviews PROSPERO (CRD42023460170), adhering to the Preferred Reporting Items for Systematic Reviews and Meta-analyses (PRISMA) guidelines [14]. This meta-analysis neither includes any attempts to obtain unpublished data, nor modifies the methodology described in the clinical trials under analysis, and thus does not use patient data; hence, we did not attempt to obtain informed consent. Since this study is a review that uses published data from the included studies, the Institutional Review Board waived the requirement for ethical approval.

### 2.2. Search Strategy

A meticulous bibliographic search of studies between 2017 and 2023 from electronic databases like MEDLINE via PubMed, EMBASE; the Cochrane Central Register of Controlled Trials (CENTRAL) in the Cochrane Library; and the Cumulative Index to Nursing and Allied Health Literature was undertaken in this study.

A bibliographic search of studies using MeSH subject heading was carried out utilizing search terms related to the subject, including GR volume, feeding intolerance, and preterm infants, with Boolean operators and truncation employed as a search strategy, screening Medline, CINHAL, the Cochrane Library, and Embase databases. Table 1 explains the search strategy for the databases explored and yjr keywords used for assimilating articles to review. The reference sections of retrieved original articles and reviews were further scanned for studies that might have been missed in the primary search.

### 2.3. Study Selection

Relevant full text articles were collected after reviewing the titles and abstracts, based on the predesigned criteria guided by the PICO framework for a systematic review and meta-analysis, as follows:

### 2.4. Population

Preterm neonate (<37 weeks’ gestation) infants on gavage feeds.

### 2.5. Intervention

Routine prefeed gastric residual monitoring.

### 2.6. Comparator

No prefeed gastric residual aspiration (or alternative intervention).

### 2.7. Outcome

At least one clinical outcome should be reported, such as the time to reach full enteral feeds, number of parenteral nutrition days, sepsis, mortality, etc.

### 2.8. Inclusion and Exclusion Criteria

Table 2 below presents the eligibility criteria for this study.

**Figure 1:**
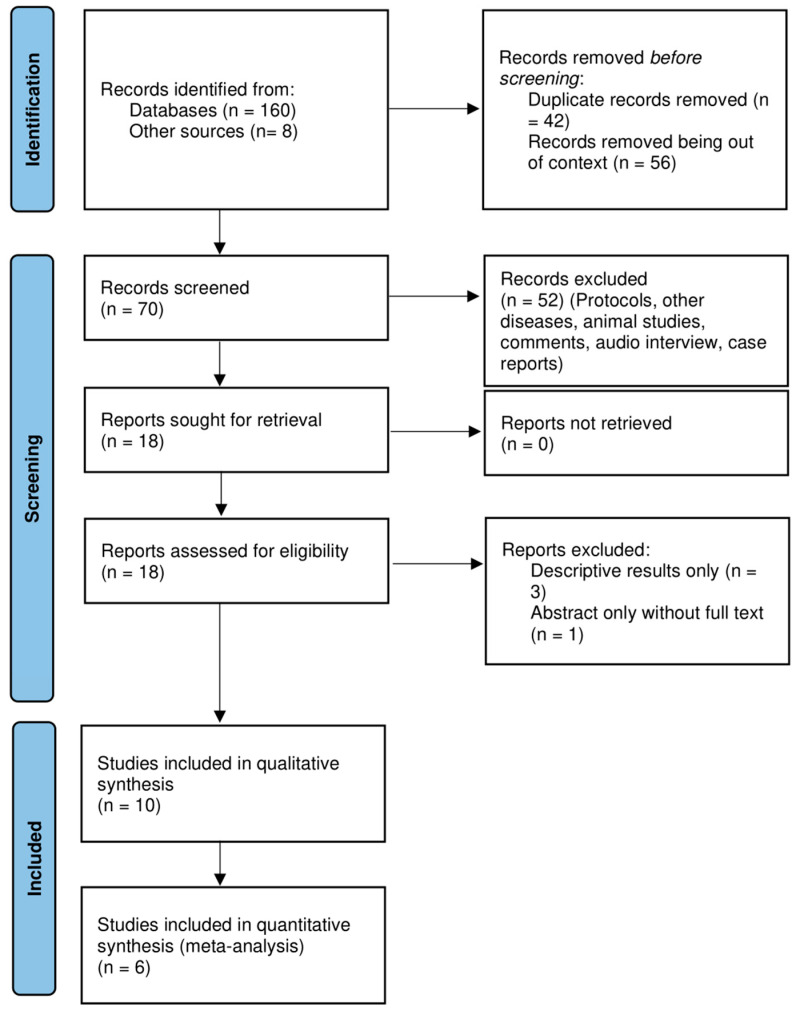
Prisma flow diagram of the inclusion of studies.

Figure 1 illustrates the PRISMA flow diagram [15] of the selection process in sufficient detail for the studies thus selected.

No language restrictions were used, and translations were used where appropriate. Empirical articles, studies with the outcomes of interest in this study not analyzed, letters to editors, editorials, abstracts without the full text, duplicates of articles, and animal studies were excluded. Review manager 5.4.1 (Revman, Cochrane Collaboration, and Oxford, UK) was used to analyze the data for this study.

### 2.9. Data Collection and Analysis

The titles and abstracts were screened for eligible articles. Only those abstracts that fulfilled the selection criteria were chosen for the full-text review. Any disagreements concerning studies’ eligibility were discussed with an expert in the field and resolved. After that, duplicates were excluded. Reference lists of the retrieved studies were also screened for relevant articles. The retrieved full-text studies were checked to confirm whether they met the inclusion criteria. The standard methods of Cochrane Neonatal [16] were used for guidance for data collection in this review. A spreadsheet data collection form was designed to extract and enter the relevant data fields from the selected full-text studies. Data were extracted into a spreadsheet which was custom-designed with the information on methodology, study participant’s characteristics, outcomes addressed in the study, details of the intervention and control group, and remarks on each included study.

### 2.10. Assessment of Risk of Bias in Included Studies

The risk of the bias of all of the included trials was assessed using the Cochrane Collaboration tool for assessing the risk of bias (ROB) [17], with studies graded as having a low, unclear, or high risk of bias. Using the Grading of Recommendations, Assessment, Development and Evaluations (GRADE) [18], the quality of evidence was rated as high, moderate, low, or very low, as described in Table 3.

Using a funnel plot [19], the risk of publication bias was visually evaluated. The results of this are incorporated with the tabular description of characteristics of the included studies in Table 4.

**Figure 2:**
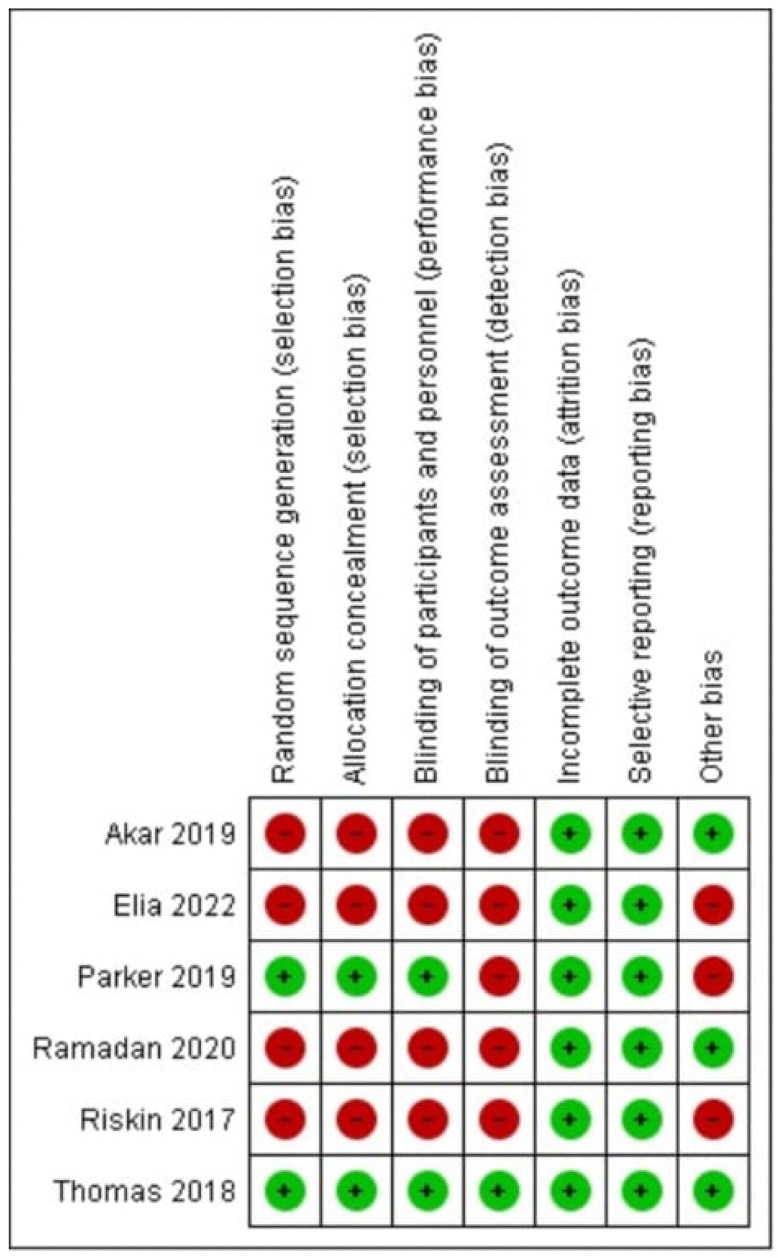
Risk of bias summary: review author’s judgements about each risk of bias item for each included study [20,21,22,23,24].

Figure 2 is an illustrative depiction of the author’s judgment using the ROB tool, version 2.

### 2.11. Data Analysis

The primary outcomes used to evaluate the efficacy of the intervention were the incidence of NEC stage 2 or higher according to modified Bell’s staging [26] and the time taken to obtain full enteral feeds (150 or 180 mL/kg/day). The number of days of parenteral feeding, length of hospital stays, and incidence of late sepsis were the secondary outcomes used to evaluate the safety of the intervention. The length of time it took to regain birth weight, the number of days spent using a central venous line, the feed intolerance events that necessitated stopping feeding, the anthropometry data at discharge and at 40 weeks postmenstrual age, and all-cause mortality were the other outcomes that were taken into account.

### 2.12. Statistical Analysis

For outcomes for which pertinent data were available from the included studies, a meta-analysis was carried out. The mean difference (MD) was computed for continuous variables and the pooled risk ratio (RR) for dichotomous variables using the Mantel-Haenszel technique. As recommended by the Cochrane manual, standard deviations were determined by applying the appropriate statistical conversion procedures to interquartile ranges, ranges, or 95% confidence intervals (CIs) [27]. The untransformed value was used as the mean when the results were given as least square means (LSMs). Sensitivity analysis was used to investigate the potential for heterogeneity resulting from variations in the computation of the arithmetic mean and LSM, and standardized mean differences were employed when suitable for continuous outcomes.

### 2.13. Measures of Treatment

The reported RR and risk difference (RD) for dichotomous data, and mean difference (MD) for continuous data, with respective 95% Cis, were used for the analysis of the treatment effects in the included studies with RevMan.

### 2.14. Assessment of Heterogeneity

Visual inspection with forest plots was used to investigate and study heterogeneity, and statistical tests such as the Chi-square test on Cochrane’s Q statistics and the I^2^ statistic were used to quantify it. The categories for heterogeneity were as follows: none (less than 25%); low (25% to 49%); moderate (50% to 74%); and high (more than 75%) [28]. When moderate or high heterogeneity (I ≥ 50%) was detected, an additional investigation into the potential causes—such as alternative research design strategies, participant demographics, interventions, or the thoroughness of outcome assessments—was conducted.

### 2.15. Subgroup Analysis

As a subgroup analysis is a useful tool for identifying potential sources of variation in the treatment effect, this study included a subgroup study based on birth weight (<1000 g, 1000 g to 1499 g, ≥1500 g), gestational age (≤27 weeks, 28 weeks to 31 weeks, ≥32 weeks), and any other interventions in the control group, wherever applicable.

## 3. Results

### 3.1. Study Search

A total of 160 records along with 8 additional records were identified from the initial electronic database search. A total of 70 records were screened after filtering the duplicates and irrelevant articles. A total of 18 studies were considered eligible for full- text screening. Only six articles were deemed suitable for quantitative synthesis (Figure 1).

The six included trials [20,21,22,23,24,25] had a total of 1118 participants, with 592 participants with routine prefeed gastric residual monitoring compared to 526 participants without the routine practice of gastric residual monitoring (n = 26) with an alternative intervention of abdominal circumference monitoring [25]. Table 4 displays the characteristics of these included studies. The blinding of participants and personnel was not implemented in any article except one [22]; consequently, there was a significant possibility of performance bias. Likewise, blinding for outcome evaluation was implemented in only one trial [22]. Because of the variability in study populations, the methods used to compare interventions, and the variability in outcome reporting units, there was a high risk of detection bias for subjective outcomes—such as time to reach full enteral feeds, feed intolerance, withholding feeds, and length of hospital stay—in nearly all the included studies (Figure 1). The PRISMA flow diagram depicts the results of the search conducted in this study. The results of the risk of bias in the included studies are illustrated in Figure 2.

### 3.2. Primary Outcomes

**Figure 3:**
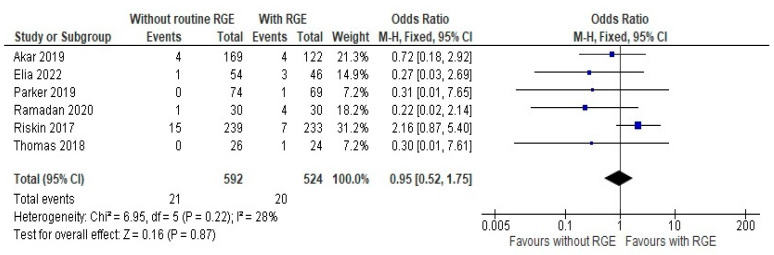
Forest plot of comparison: the incidence of NEC stage 2 or more [20,21,22,23,24,25].

The primary outcomes assessed in this study involved making a comparison between the two groups: the incidence of NEC stage 2 or more and the time achieve full feeds of at least 150 mL/kg or more. The comparison of the occurrence of NEC between the experimental (with the routine gastric residual evaluation) and control group (without gastric residual evaluation or alternative methods) from the six studies which were pooled in the meta-analysis [20,21,22,23,24,25] showed a mean difference of 0.95 (95% CI: 0.52, 1.75) using a random-effects model with inverse variance (IV) weighting. Heterogeneity was low (Chi^2^ = 6.95, df = 5, *p* = 0.22; I^2^ = 28%), and the overall effect was not statistically significant (Z = 0.16, *p* = 0.87), as seen in Figure 3. It is worth mentioning that studies with a small sample size are weighted much higher than studies with larger sample size due to the narrower confidence interval.

**Figure 4:**
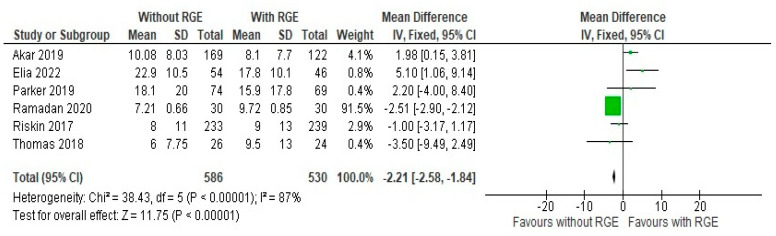
Forest plot of comparison: time taken to establish full enteral feeds ≥ 150 mL/kg/d (days) [20,21,22,23,24,25].

A volume of at least 150 mL/kg or more as the time to the full feeds was taken for comparison, as the definitions of defining feed intolerance and threshold of stopping feeds were not consistent across the studies. The results of the comparison of the time taken to achieve full enteral feeds between the groups were expressed as a mean difference of −2.21 (95% CI: −2.58, −1.84). Pooled studies showed heterogeneity (Chi^2^ = 38.43, df = 5, *p* < 0.00001; I^2^ = 87%), and the overall effect was statistically significant (Z = 11.75, *p* < 0.00001), as seen in Figure 4.

This suggests that the avoidance of the practice of gastric residual monitoring can lead to a shorter duration to achieve an optimal feed goal for the preterm infant, once again pointing out the lack of efficacy of the practice. Thus, the results of the comparison of primary outcomes points out the minimal efficacy of the intervention and the evidence supports the avoidance of the practice.

### 3.3. Secondary Outcomes

**Figure 5:**
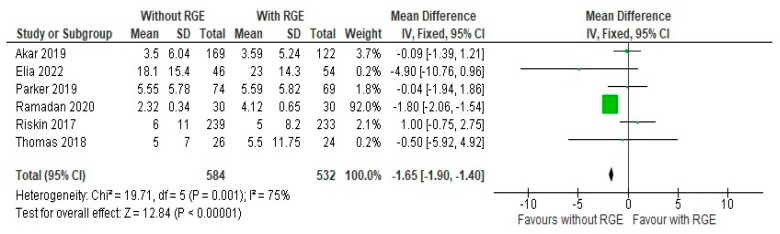
Forest plot of comparison: number of days of parenteral nutrition [20,21,22,23,24,25].

All the included studies reported both the number of days of parenteral nutrition, the duration of hospital stay (days), and the incidence of late sepsis, and these were included in this meta-analysis for comparison between the two groups. On comparing the number of days of parenteral nutrition between the experimental and control group, a mean difference of −1.65 (95% CI: −1.90, −1.40) was identified. Pooled studies showed heterogeneity (Chi^2^ = 19.71, df = 5, *p* = 0.001; I^2^ = 75%), and the overall effect was statistically significant (Z = 12.84, *p* < 0.00001), as seen in Figure 5. This points to the conclusion that it is safe to avoid gastric residual monitoring as it can significantly reduce the duration of parenteral nutrition.

**Figure 6:**
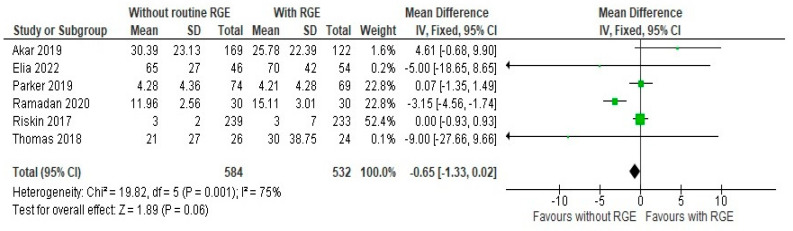
Forest plot of comparison: duration of hospital stays (days) [20,21,22,23,24,25].

The comparison of the duration of hospital stays (days) between the groups showed a mean difference of −0.65 (95% CI: −1.33, 0.02). Pooled studies showed heterogeneity (Chi^2^ = 19.82, df = 5, *p* = 0.001; I^2^ = 75%) and the overall effect was not statistically significant (Z = 1.89, *p* = 0.06), as seen in Figure 6, suggesting moderate support for avoiding the intervention, as it can reduce the duration of hospital stay.

**Figure 7:**
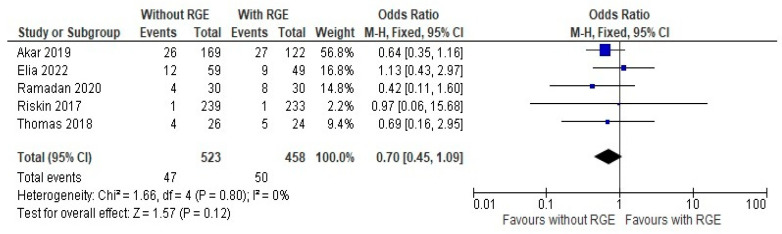
Forest plot of comparison: incidence of late sepsis [20,21,23,24,25].

The comparison of the incidence of late sepsis between the groups showed a mean difference of 0.70 (95% CI: 0.45, 1.09). Pooled studies did not show heterogeneity (Chi^2^ = 1.66, df = 4; *p* = 0.80; I^2^ = 0%), and the overall effect was not statistically significant (Z = 1.57; *p* = 0.12), as seen in Figure 7. Moderate evidence is generated here favoring the avoidance of gastric residual monitoring, due to the chance of developing late sepsis. It can be deemed from these results that comparisons of the secondary outcome assessments, avoiding gastric residual monitoring, are safe for preterm infants.

### 3.4. Other Outcomes

From the included studies [20,21,22,23,24,25], the number of days of central venous line (CVL) usage, episodes of feed intolerance requiring stopping feeds, anthropometry data [22] at discharge and 40 weeks postmenstrual age [24,25], and all-cause mortality before discharge were compared. Three studies [22,23,25] did not observe any significant difference in the number of times the feeds were withheld, whereas one study [25] reported significantly higher feed intolerance episodes (80% vs. 35%, *p* < 0.001) and feed interruption days (*p* < 0.001) in the routine prefeed gastric aspiration group. In one study [24], similarly, the episodes of abdominal distension were significantly higher (*p <* 0.001) in the routine prefeed gastric residual aspiration group [21]. Two trials studied enteral intake at weekly intervals, [22,25] and a study [25] compared enteral intake and concluded that there was no statistically significant difference between the groups in terms of the duration of total parenteral feeding. On the contrary, it was observed [22] that the infants who did not undergo GR evaluation had improved enteral nutrition; improved weight gain in infants reduces both intensive care unit and hospital stay. Studies [20,21,22,23,24] observed that the avoidance of routine GR control before each feeding shortens the time to full enteral intake without increasing the incidence of NEC and the duration of parenteral feeding.

### 3.5. Subgroup Analysis

A subgroup analysis was undertaken based upon weight, gestation, and the additional intervention (abdominal girth (AG) monitoring [25]). Out of six trials, three trials [21,22,24] registered infants ≤ 32 weeks/1250 g, one registered > 1500 g, whereas the remaining two registered wider ranges of gestation (27–36 weeks) and weights (750–2000) without distinct subgroup analysis. Therefore, only four trials [20,23,24,25] were available for gestation/weight subgroup analysis (<1250 g versus ≥1250 g) and these studies compared routine prefeed gastric aspiration versus avoiding routine gastric aspiration (Table 5).

There was no significant effect of gestation and weight for any of the clinical outcomes. A meta regression analysis to assess the effect of gestational age over the magnitude and direction of the effect size could not be performed due to the low number of studies (<10).

Except for time to reach full enteral feeds, there was no significant effect of additional AG monitoring in the no gastric aspiration group (Table 6).

Feed volume, rather than AG, was found to likely be related to defining full enteral feeds, as both studies of the AG group considered full enteral feeds at higher volumes (150 and 180 mL/kg) as compared to the other subgroup.

### 3.6. Sensitivity Analysis

A sensitivity analysis was undertaken based on the risk of bias in the included studies, and this was seen due to the non-blinding of participants and outcomes. The heterogeneity due to a study [24] was addressed by performing a post hoc sensitivity analysis. Removing this study from the analysis did not significantly affect the overall direction of the outcomes, although the effect size decreased, as this study had a bigger sample of participants. The standardized mean differences were calculated for the continuous outcomes included in this study. In this post hoc analysis, it was seen that the results for the du ration of hospital stay remained unchanged (−0.65 [−1.33, 0.02]), along with the duration of parenteral nutrition (−1.65 [−1.90, −1.40]) and the incidence of late sepsis (0.70 [0.45, 1.09]).

## 4. Discussion

The moderate quality evidence for the efficacy assessment of the intervention, generated from this meta-analysis of six randomized controlled trial (RCT)s, underscored the recommendation to avoid the practice of routine prefeed gastric residual aspiration, as it was observed to not be associated with an increased incidence of NEC (stage 2 or more) and to have led to an earlier achievement of full enteral feeds. A lower incidence of late sepsis, shorter duration of TPN, and an earlier discharge from the hospital, supported by low-quality evidence, suggests that avoidance of this intervention practice can be considered a safe practice.

The evidence available in this study suggests that evaluating GR does not protect against NEC in extremely preterm infants. In the past, it was assumed that the presence of an abnormally large GR volume (GRV) could be an early indicator of NEC, based on previous studies [29,30]. The relationship between GRV and NEC was examined in a case–control study, which found that the infants who developed NEC had a maximum GRV of 4.5 mL as compared to 2 mL in the control group [31]. The previous literature suggested that a higher risk for NEC was associated with a GRV greater than 3.5 mL or one-third of the previous feed volume [32].

One retrospective study [24] enrolled 472 participants and concluded that avoiding routine prefeed aspiration was associated with the earlier attainment of full enteral feeds without increasing the risk for NEC. The undesirable effects on the nutritional intake of the preterm neonate have been highlighted in the previous literature [33,34], and these observations were further supported and upheld by other prospective randomized controlled trials [35,36]. One study remarked that routine prefeed aspiration led to the overgrowth of pathogenic bacteria [37,38].

In comparison to the standard gastrointestinal evaluation for preterm neonates, there is less evidence to support the use of the AG measurement to determine clinical outcomes. This is mostly because the sample size is limited and there is a significant likelihood of variation from both intra- and inter-observer variation. Further hindering the adoption of any clinically significant cutoff value is the fact that studies have demonstrated that AG is prone to change in value during a feeding cycle, even among healthy premature infants [39]. This review recommends against routinely monitoring and evaluating stomach aspiration and saving this for newborns who exhibit clinical indications of feed intolerance and NEC.

## 5. Limitations

This review identifies several limitations which are important to acknowledge when interpreting the results and considering future research. The included trials were generally small and lacked adequate statistical power to detect significant differences in serious clinical outcomes such as NEC and mortality, and this limited the ability to draw strong conclusions from the data. Many of the trials included were at high risk for performance and detection bias, suggesting that there may be flaws in the study design or execution that could introduce bias into the results, affecting the overall certainty of the evidence.

Wide variations were observed in the feeding protocols used in the included trials, introducing confounding variables and making it difficult to isolate the effects of formula milk on adverse outcomes like NEC and feed intolerance. Future RCTs should adopt uniform study designs to minimize variability in the interventions and ensure consistency across trials, as these variations can introduce heterogeneity into the data, making it challenging to compare and generalize the results effectively.

Another drawback observed was that none of the trials provided separate data for infants with perinatal asphyxia and intrauterine growth restriction, and this lack of subgroup analysis made it impossible to determine whether the effects of prefeed aspiration differed for these specific populations. No data concerning long-term growth and neurodevelopmental outcomes were available in all the included studies. These long-term outcomes are crucial for understanding the overall impact of prefeed aspiration practices on infant health and development. Overall, these recommendations highlight the need for more rigorous and standardized research in this area to better understand the impact of routine prefeed aspiration on neonatal health outcomes.

## 6. Conclusions

This study concludes that the practice of evaluating GR is unnecessary, and omitting this practice can help preterm infants to receive more enteral nutrition without an increase in adverse health outcomes. This study highlights that those infants who did not undergo GR evaluation were not exposed to the risk of developing NEC and the incidence of late sepsis and had a shorter stay in hospital. Therefore, it seems worthwhile to forgo the practice of routine prefeed gastric residual monitoring if other signs of feed intolerance in preterm low birth weight neonates are not present.

## Figures and Tables

**Table 1:** Keyword strategy used in the database search.

Database	PICO	MeSH Term	Keyword Term
PubMed, CINAHL, Web of science, ProQuest Dissertations Cochrane, Clinical Trial Registry of India, ClinicalTrials.gov, Australian New Zealand Clinical Trial Registry, and EU Clinical Trials Register (the Cochrane Neonatal search strategy for specialized register).	Population	Preterm neonate, premature born	Preterm AND Neonate Low birth weight AND premature Preterm neonate NOT Term neonate
	Intervention	Routine prefeed gastric residue monitoring	Gastric residual OR Gastric aspirate
	Comparison	No prefeed gastric residue aspiration (or another intervention)	Avoid AND gastric residual OR abdominal Girth monitoring
	Outcome	NEC, time to reach full enteral feeds, number of days of parenteral nutrition, sepsis, and mortality	NEC AND gastric residual monitoring time to reach full enteral feeds AND gastric residual monitoring, sepsis AND gastric residual monitoring, mortality AND gastric residual monitoring
	Time	2017–2023	
	Study	Randomized OR quasi-randomized trials OR cluster-randomized trials	

NEC: necrotizing enterocolitis, CINAHL: Cumulative Index to Nursing and Allied Health Literature.

**Table 2:** Eligibility criteria for study selection in this study.

Criteria	Inclusion Criteria	Exclusion Criteria
Type of studies	Randomized or quasi-randomized trials and cluster-randomized trials from 2017 to 2023, comparing routine prefeed gastric residual aspiration with either no aspiration or any other intervention.	Reviews, letters to editors, editorials, survey reports, only abstract available, animal studies
Type of participants	Preterm (<37 weeks’ gestation) infants on gavage feeds (nasogastric (NG) tube).	Term neonates, older children
Type of intervention and control	Intervention: Routine monitoring of gastric of enteral feeds in infants.	Studies with descriptive results and outcomes not numerically reported
	Control: No monitoring of gastric residual or other alternative monitoring approach.	Studies reporting laboratory data only
Article	Full text availability, all language articles.	

**Table 3:** GRADE categories of quality of evidence.

GRADE Quality of Evidence	Interpretation
High quality	“Further research is very unlikely to change the confidence in the estimate of effect”
Moderate quality	“Further research is likely to have an impact confidence in the estimate of effect and may change the estimate”
Low quality	“Further research is very likely to have an impact confidence in the estimate of effect and is likely to change the estimate”
Very low quality	“Any estimate of effect is very uncertain”

**Table 4:** Characteristics of the included studies with results of GRADE.

Study Authors	Participant Characteristics				Number of Patients		Outcome/s Assessed in the Study	Remarks	GRADE Evidence
					Routine Gastric Residual Volume Evaluations	No Gastric Residual Volume Evaluations or Other Interventions Adopted			
	Gestational Age (Week) (SD)	Birth Weight (g) (SD)	Gestational Age (Week) (SD)	Birth Weight (g) (SD)					
Akar 2019 [20]	30.37 ± 2.58	1538.48 ± 509.05	29.31 ± 3.37	1443.65 ± 550.38	169	122	-Days to full enteral intake; -Days of parenteral nutrition; -Sepsis (late sepsis); -NEC ≥ grade 2, patent ductus arteriosus; -IVH (all grades); -Duration of invasive mechanical ventilation (days); -Weight at discharge (g); -Duration of nCPAP (days); -Duration of hospitalization (days).	Total duration of parenteral nutrition, ≥grade 2 NEC, weight at discharge and duration of hospitalization were reported to be similar between the groups. Supports the avoidance of routine gastric residual control before each feeding as it shortens the time to full enteral intake without increasing the incidence of NEC and duration of parenteral feeding.	Moderate
Elia 2022 [21]	28.1 ± 2.5	1084 ± 347	27.8 ± 2.2	1100 ± 427	59	49	-Age at full (150 mL/kg/d) enteral feeding (d); -Age at full oral enteral feeding (d); -Age at birth weight recovery (d); -Duration of parenteral nutrition (d); -Duration of NICU stay (d); -Duration of hospital stay (d).	Supports the selective monitoring of GR in extremely preterm infants and was associated with a decrease in age at full enteral feeding and at birth weight recovery, and was associated with better Z-scores of weight at discharge in comparison with routine GR monitoring.	Moderate
Parker 2019 [22]	27.1 (2.4) (SD), wk	888.8 (206.6) (SD), g	27.0 (1.2) (SD), wk	915.2 (180.0) (SD), g	74	69	-Days to full feeds, 120 mL/kg/d; -Hours of parenteral nutrition; -Hours with central access; -Days requiring invasive ventilation; -Days to discharge.	Incidence of NEC, late-onset sepsis, and ventilator-associated pneumonia were reported to be similar between groups. The study concluded that among extremely preterm infants, the omission of gastric residual evaluation increased the delivery of enteral nutrition as well as improved weight gain and led to earlier hospital discharge.	Moderate
Ramadan 2020 [23]	32.13 ± 3.34	75.19 ± 12.89	32.68 ± 2.99	74.88 ± 11.56	30	30	-Days to full enteral intake of 120 mL kg per day; -Days of PN; -Days of life to parenteral nutrition discontinuation; -Days to discharge; -Sepsis; -NEC (definite); -Weight at discharge mean.	It is concluded here that eliminating routine prefeed gastric aspirate monitoring decreases late-onset sepsis, permits preterm infants to receive complete enteral feeds sooner, and allows them to leave the hospital sooner. It also did not affect the likelihood of mortality or NEC.	Low
Riskin 2017 [24]	32.0 (29.7–33.0)	1625 (1207–1934)	32.4 (30.4–33.4)	1645 (1297–1954)	239	233	-NPO (d); -Number of NPO episodes; -PN (d); -Age at full enteral feeds (d); -Time to full enteral feeds (d); -Age at full PO feeds (d); -LOS (d); -Weight at discharge (g); -Weight gain from birth to discharge (g); -Percent weight gain (%); -Postmenstrual age at discharge (wk); -NEC (%) infants with NEC Bell stage ≥ 2 (%); -Days of antibiotic treatment (d); -Number of infections.	Reports that the time to full oral feedings and lengths of stay were similar in both groups. The rate of NEC was reported as being higher in the selective gastric residual volume evaluation group. This study states that the strongest predictor of time to full enteral feedings is GA. This study highlights that the routine evaluation of gastric residual volume and increasing time on non-invasive ventilation both prolong the attainment of full enteral feedings.	Moderate
Thomas 2018 [25]	30.8 ± 1.5	30.8 ± 1.5	30.8 ± 1.5	1312 ± 265.7	24	26 (AG monitoring)	-Time to reach full feeds, d; -Episodes of feed intolerance; -No. of feeds withheld; -Duration of hospital stay, d; -Duration of parenteral nutrition, d; -Sepsis, n; -NEC.	This study concludes that the infants in the AG group reached full feeds earlier than infants in the GRV group. No significant differences were found between the two groups with regard to secondary outcomes. The study recommends abdominal girth measurement as a marker for feed tolerance but suggests that it needs to be studied in infants less than 750 g and at less than 26 weeks of gestation.	Low

AG abdominal girth monitoring; GR gastric residual; IVH intraventricular hemorrhage; LOS length of stay; nCPAP nasal continuous positive airway pressure; NEC necrotizing enterocolitis; NICU Neonatal Intensive Care Unit; NPO Nothing by Mouth; PN parenteral nutrition; PO per os (orally).

**Table 5:** Comparison of other secondary outcomes (avoiding routine gastric aspiration versus routine prefeed gastric aspiration).

Outcome	No. of Studies (Participants)	RR/MD [95% CI]	Heterogeneity (I^2^), *p* Value
Time to reach full feeds (180 mL/kg/day)	4 (996)	−3.00 [−3.26, −1.52]	0%, 0.7
Any sepsis	5 (1118)	0.617 [0.32, 0.81]	0%, 0.5
Days of central venous line usage	2 (434)	−0.98 [−5.12, 1.18]	64%, 0.3
All-cause mortality	2 (160)	0.44 [0.23, 0.92]	0%, 0.2
Time to regain birth weight	1 (100)	−0.69 [−1.38, 0.88]	0%, 1.1

**Table 6:** Subgroup analysis for primary and secondary outcomes (comparing routine versus no gastric residual).

Outcome Parameter	≤32 Weeks/≤1250 g		>1250 g	
	No. of Studies (Participants)	RR/MD [95% CI]	No. of Studies (Participants)	RR/MD [95% CI]
NEC (stage 2 or more)	5 (1118)	1.00 [0.13, 3.12]	-	0.04 [0.02, 1.29]
Time to reach full enteral feeds	5 (1118)	−1.22 [−3.41, 0.22]	-	−0.01 [−1.43, 1.82]
Culture-positive sepsis	4 (1020)	0.73 [0.59, 1.31]	1 (46)	0.116 [0.02, 2.98]
Any sepsis	4 (966)	0.90 [0.52, 1.12]	1 (122)	0.16 [0.01, 1.91]
Days of total parenteral nutrition	5 (1118)	−0.23 [−1631, 0.31]	-	-
Days of central venous line usage	4 (434)	−1.37 [−5.51, 2.0]	-	-
All-cause mortality	2 (160)	0.13 [0.05, 0.34]	1 (17)	0.271 [0.01, 8.11]
Time to regain birth weight	-	-	1 (100)	−1.21 [−6.05, 1.35]
Duration of hospital stay	5 (11066)	−5.30 [−12.00, 0.33]	-	-

## Data Availability

The datasets used and/or analyzed during the current study are available from the corresponding author on reasonable request.
